# Clinical Oxidative Stress during Leprosy Multidrug Therapy: Impact of Dapsone Oxidation

**DOI:** 10.1371/journal.pone.0085712

**Published:** 2014-01-22

**Authors:** Taysa Ribeiro Schalcher, Rosivaldo S. Borges, Michael D. Coleman, João Batista Júnior, Claudio G. Salgado, Jose Luiz F. Vieira, Pedro R. T. Romão, Fabio R. Oliveira, Marta Chagas Monteiro

**Affiliations:** 1 Laboratório de Microbiologia e Imunologia Clínica/UFPA and Programa de Pós-graduação em Ciências Farmacêuticas, Faculdade de Farmácia, Universidade Federal do Pará/UFPA, Rua Augusto Corrêa, Belém, Brasil; 2 Mechanisms of Drug Toxicity Group, Department of Pharmaceutical Sciences, Aston University, Aston Triangle, Birmingham, United Kingdom; 3 Centro Universitário do Distrito Federal - UDF, SEP/SUL EQ 704/904 - CONJ A, Brasília/DF, Brasil; 4 Laboratório de Dermato-Imunologia UFPA/MC, Marituba, Pará, and Programa de Pós-graduação em Neurociências e Biologia Celular, Instituto de Ciências Biológicas, Universidade Federal do Pará/UFPA, Bairro Guamá, Belém, Brasil; 5 Programa de Pós-graduação em Ciências da Saúde. Universidade Federal de Ciências da Saúde de Porto Alegre, Porto Alegre, Brasil; Louisiana State University and A & M College, United States of America

## Abstract

This study aims to assess the oxidative stress in leprosy patients under multidrug therapy (MDT; dapsone, clofazimine and rifampicin), evaluating the nitric oxide (NO) concentration, catalase (CAT) and superoxide dismutase (SOD) activities, glutathione (GSH) levels, total antioxidant capacity, lipid peroxidation, and methemoglobin formation. For this, we analyzed 23 leprosy patients and 20 healthy individuals from the Amazon region, Brazil, aged between 20 and 45 years. Blood sampling enabled the evaluation of leprosy patients prior to starting multidrug therapy (called MDT 0) and until the third month of multidrug therapy (MDT 3). With regard to dapsone (DDS) plasma levels, we showed that there was no statistical difference in drug plasma levels between multibacillary (0.518±0.029 µg/mL) and paucibacillary (0.662±0.123 µg/mL) patients. The methemoglobin levels and numbers of Heinz bodies were significantly enhanced after the third MDT-supervised dose, but this treatment did not significantly change the lipid peroxidation and NO levels in these leprosy patients. In addition, CAT activity was significantly reduced in MDT-treated leprosy patients, while GSH content was increased in these patients. However, SOD and Trolox equivalent antioxidant capacity levels were similar in patients with and without treatment. These data suggest that MDT can reduce the activity of some antioxidant enzyme and influence ROS accumulation, which may induce hematological changes, such as methemoglobinemia in patients with leprosy. We also explored some redox mechanisms associated with DDS and its main oxidative metabolite DDS-NHOH and we explored the possible binding of DDS to the active site of CYP2C19 with the aid of molecular modeling software.

## Introduction

Leprosy, also known as Hansen's disease, is a chronic infectious disease caused by *Mycobacterium leprae*. Leprosy remains a significant public health problem in several parts of the world. According to official reports submitted to World Health Organization (WHO) by 105 countries, the number of new cases detected during the year 2011 was 219,075. Indeed, in 2012, 33,955 new cases were detected in Brazil alone [1]. In this context; several epidemiological studies have documented the main regions of Brazil with a high prevalence of leprosy [2]–[4].

The current strategy for leprosy control recommended by the WHO is based on multidrug therapy (MDT) that consists of the combination of rifampicin, clofazimine and dapsone (DDS) for multibacillary (MB) leprosy patients and rifampicin and dapsone for paucibacillary (PB) leprosy patients [1]. DDS (4,4′-diaminodiphenylsulfone) has bacteriostatic action against *Mycobacterium leprae* and is an essential component of MDT. The action of DDS is due to inhibition of dihydrofolic acid synthesis by competition with para-aminobenzoic acid (PABA) [5]. DDS distributes in all body organs including skin, liver, kidneys, and erythrocytes, and crosses the blood-brain barrier and the placenta, as well as being found in breast milk [6]. DDS was initially used as an antibiotic in humans at doses equivalent to sulfonamides, which led to severe hemolytic anemia and methemoglobinemia [7], [8].

Recently, our studies on the molecular structure/activity properties of DDS showed that its biological properties are strongly influenced by redox mechanisms associated with its sulphone group as well as its nucleophilic aniline rings. Hence, during the oxidative clearance of dapsone in man, hepatic CYPs exploit the propensity of the molecule to undergo electron transfer or oxidation to N-hydroxylated metabolites such as DDS-NHOH and monoacetyl-hydroxylamine MADDS-NHOH [7]–[9]. Thus, through its metabolically formed hydroxylated derivatives, DDS is able to exert local oxidative stress conditions which impacts macromolecules, such as proteins, lipids, carbohydrates and nucleic acid, ultimately leading to cellular necrosis in patients [7], [10]. The primary manifestation of the oxidative capacity of dapsone-related hydroxylamines, is their induction of methemoglobinemia in patients which may also lead to hemolysis [7]. Indeed, methemoglobin formation is caused by the co-oxidation of the hydroxylamine metabolites, with oxyhemoglobin in erythrocytes [7], [10]. In this study, we investigated the contribution of multidrug therapy, which includes dapsone, towards the generation of oxidative stress and cell damage through the analysis of antioxidant status (total antioxidant capacity, superoxide dismutase and catalase activities), oxidative markers (nitric oxide levels, lipoperoxidation, methemoglobin formation) and DDS levels in patients with leprosy. The results were then associated with the known redox mechanisms DDS and DDS-NHOH, through molecular modeling studies. Whilst the role of the hydroxylamine metabolites in dapsone toxicity is well established, the CYP isoforms primarily responsible for their formation have been the subject of considerable study in a variety of clinical and experimental models over past decades; indeed, CYP3A4, CYP2E1 and CYP2C9 [11]–[13] have each been postulated as the major contributor to the oxidation of this drug. Latterly, a role for CYP2C19, has been outlined in a study with recombinant isoforms [14] and in our report we also explore the potential interactions between dapsone and CYP2C19 using molecular docking analysis.

## Methods

### Ethics statement

The Ethics Committee of the Federal University of Pará, Brazil, approved the study protocol (protocol 079/09). It was also approved by the State Reference Unit for Leprosy Treatment Dr. Marcelo Candia, Marituba and Health unit Guama, Brazil and so gave permission to start collecting data. All participants were informed about the aims and methods of study and they also wrote and signed the informed consent before the start of the experiment and sample collection.

### Population and experimental design

In this study, a total of forty-three subjects comprising twenty-three patients diagnosed with leprosy, receiving care in the Department of the State Reference Unit for Leprosy Treatment under Dr. Marcello Candia- Marituba, and in the Health Unit of Guamá, Belém, Para, Brazil. These patients were selected for the study before starting multidrug therapy (called MDT 0) and they were followed until the third month of multidrug therapy (MDT 3). Leprosy patients (age range, 20–45 years) were classified into paucibacillary (PB; 9 cases) and multibacillary (MB; 14 cases), based on WHO clinical criteria (testing positive for 2 of the 3 clinical criteria—skin lesions (≤5, PB and >5, MB), anesthesia, and nerve enlargement) and Bacteriological Index (BI) [1]. Two slit-skin smears, one from a representative lesion and the other from an earlobe, were obtained, and then stained using modified Ziehl–Neelsen technique. A minimum of 100 oil-immersion fields of the smear were examined for the presence of acid-fast bacilli, and the BI was calculated.

Leprosy patients with reactions, ulceration, a history of smoking, or those under the influence of alcohol, over 45 years of age, with co-infections or diabetes mellitus or other systemic diseases or health problems, and history of drug and nutraceutical use, including vitamins, ascorbic acid, and tocopherol, were excluded to rule out their possible influence on the study parameters. In this study, patients were divided into two groups: samples collected before administering the first dose MDT-supervised (MDT 0) and after two months in the third dose MDT-supervised (MDT 3).

Healthy adults were selected voluntarily to serve as controls (n = 20). This group did not have signs and symptoms of leprosy, other diseases, or health problems, and the volunteers were nonsmokers and free from drug use. The control group consisted of sex-matched individuals, aged 20–45 years, who were living in the same settings as those of the patients.

**Figure 1:**
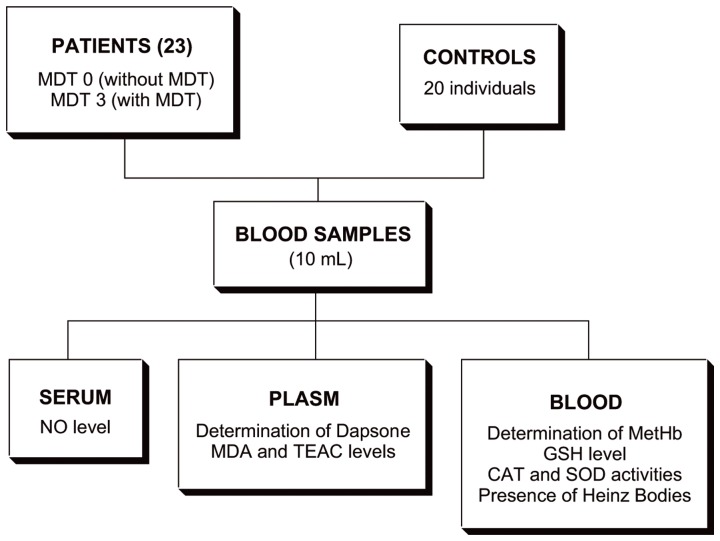
Sampling procedure.

Blood samples (10 mL) were obtained from all participants by venipuncture in tubes containing ethylenediaminetetraacetic acid (EDTA). The whole blood samples were divided into two aliquots; the first was used immediately for determining of MetHb and GSH levels, activities of SOD and CAT and presence of Heinz Bodies, whilst the second aliquot was centrifuged at 2000× g for 6 min, to separate plasma for the analyses of MDA and total antioxidant status (TAS) levels as well as dapsone concentration analysis. In addition, the serum was collected for NO measurement (Figure 1).

### DDS determination

The plasma concentrations of DDS were determined in accordance with the procedures of extraction, quantification and standardized by Kwadijk and Toraño [5], using High Performance Liquid Chromatography (HPLC). The model used in the chromatograph was ProStarVARIAN work with ODS RP18 column, 25 cm×4 mm, UV detection at a wavelength of 295 nm and flow 1 mL/min. As mobile phase we used aqueous solution of 30% acetonitrile and as internal standard, phenacetin solution of 100 µg/mL.

### Determination of Methemoglobin (MetHb) Content

MetHb content was evaluated from the change in absorbance at a wavelength of 632 nm; this change was caused by addition of potassium cyanide (KCN) to the buffered hemolysate. A dilution of the hemolysate, in which potassium ferricyanide (K_3_Fe(CN)_6_) was used to convert all possible forms of hemoglobin (Hb) to MetHb, was used as a reference solution. The MetHb content was measured in duplicate, and values less than 2% were considered normal [15].

### Determination of Lipid Peroxidation

Lipid peroxidation was measured by quantifying MDA in blood samples of patients using the thiobarbituric acid-reactive substances (TBARS) assay. This method is a very useful, economical, and easy-to-use assay for evaluating oxidative stress [16]. Briefly, lipoproteins were precipitated by addition of samples to 0.05 M trichloroacetic acid (TCA) and 0.67% TBA in 2 M sodium sulfate. The union of lipid peroxide and TBA was performed by heating in a water bath for 90 min. The chromogen formed was extracted in n-butanol, which was measured at a wavelength of 535 nm. Lipid peroxidation was expressed as nanomoles of MDA per liter.

### Measurement of Total Antioxidant Status

The total antioxidant status (TAS) is a sensitive and reliable marker to detect in vivo oxidative stress changes that may not be detectable through the measurement of a single, specific antioxidant. In this study, the TAS was evaluated by Trolox equivalent antioxidant capacity (TEAC). In this assay, 2, 2-azino-bis (3-ethylbenzothiazoline, 6-sulfonate) (ABTS2) is incubated with persulfate to produce ABTS+. This species is blue-green. Antioxidants present in the sample cause a reduction in absorption proportional to their concentration. The antioxidant capacities of the samples are expressed as TEAC using a calibration curve plotted with different amounts of Trolox and their absorbance measured at 740 nm [17].

### Determination of Nitric Oxide (NO)

The nitrate (NO_3_^−^) present in the serum samples obtained from the patients was reduced to nitrite using nitrate reductase, and the nitrite concentration was determined by the Griess method [18]. Briefly, 100 µL of the serum supernatant was incubated with an equal volume of the Griess reagent for 10 min at room temperature. The absorbance was then measured on a plate scanner (Spectra Max 250; Molecular Devices, Menlo Park, CA, USA) at 550 nm. The nitrite (NO_2_^−^) concentration was determined using a standard curve generated using sodium nitrate (NaNO_2_). Nitrite production is expressed per µM.

### Glutathione (GSH) Levels

Determination of the intracellular GSH levels was based on the ability of GSH to reduce 5,5-dithiobis-2-nitrobenzoic acid (DTNB) to nitrobenzoic acid (TNB), which was quantified by spectrophotometry at 412 nm. The methodology described by Ellman [19] was adapted for this determination, and GSH concentrations were expressed in µmol/mL. This assay was adapted for use in a microtitre plate using a microplate spectrophotometer system, Spectra MAX 250 (Molecular Devices, Union City, CA, USA) [20].

### Superoxide Dismutase (SOD) Activity

Determination of SOD activity was performed according to the procedure recommended by McCord and Fridowich [21]. This method evaluated the ability of SOD to catalyze the conversion of O_2_^−^ to H_2_O_2_ and O_2_. SOD activity was measured using UV spectrophotometry at a wavelength of 550 nm and was expressed in nmol/mL.

### Catalase (CAT) Activity

Determination of CAT activity was performed according to the procedure recommended by Bleuter [22]. We evaluated the ability of the enzyme present in the sample to convert H_2_O_2_ to H_2_O and O_2_. The decay of H_2_O_2_ was measured using ultraviolet spectrophotometry at 240 nm, and CAT values were expressed as units per gram of hemoglobin (U/g protein).

### Presence of Heinz Bodies

The precipitation of protein aggregates from the cytoplasmic membrane produces so-called Heinz bodies. Such inclusions can be observed under an optical microscope by staining with crystal violet or brilliant cresyl blue and visualized as globular inclusions of approximately 3 µm. Laboratory analysis for the quantification of Heinz bodies was based on the methodology employed by Rimiolli and Godoy [15].

### Statistical Analysis

Data are reported as the mean ± SD values. Statistically significant differences between groups were determined using Analysis of Variance (ANOVA) followed by Tukey multiple comparison tests. P<0.05 was considered statistically significant.

### Molecular Docking

For studies of the interactions involving dapsone with the biological macromolecule human microsomal cytochrome P450 (CYP) 2C19, we conducted a computer assisted virtual molecular docking [23], which is one of the techniques of molecular modeling, which consists of: predicting the bioactive conformation of a micromolecule (ligand) at the site of a biological macromolecule, followed by the evaluation and classification of the proposed binding mode [24]. The structure of the human microsomal CYP2C19 (4GQS, resolution 2.87 Å) obtained from the crystallographic protein data bank (PDB) and used in the study of docking was resolved experimentally by X-ray crystallography [25]. Prior to the study of virtual molecular docking, molecular mechanics calculations were performed with the three-dimensional structure developed for dapsone, based on the force-field MMFF94 [26], so that there would be a better accommodation of the free atoms in the respective structure of dapsone. Computational tools for the performing of the virtual molecular docking differ in the manner of dealing with the flexibility of the enzyme, the ligand, its algorithm and scoring function [27]. In the present study, the molecular docking algorithm chosen was MolDock [23]. The study was performed in the flexible docking mode, using the scoring function of the docking algorithm, which is an extension of the linear potential by parts (PLP), the spatial adjustment of a wide variety of conformations of dapsone were tested at the catalytic interaction site of CYP2C19 and several docking solutions were generated automatically from the input structure of dapsone. The accuracy of the docking was further improved by using the algorithm rescoring function, which identified the most promising docking solution among the solutions obtained by the docking algorithm. We examined the complementarity of the interactions of the CYP2C19-DDS complex formed and identified the results that provided the best correlation between the poses and scores obtained.

## Results and Discussion

### Demographic and laboratorial characteristics of patients with leprosy

The final study cohort comprised 23 patients and 20 healthy individuals (control). Of these patients, 39% were classified as PB and 61% as MB. The twenty-three patients who initiated this research before MDT (called MDT0) were followed up until the third supervised dose of MDT (called MDT3). Most patients were female (54%) with age varying from 20–30 years (55%), 29% had a positive bacteriological index (BI) varying from degree 0.1–6, 39% presented disability (1 or 2 scores), and 28% had nerve damage (1 to 3 scores). All healthy participants did not show BI, degree of disability, or nerve damage (Table 1).

**Table 1:** Demographic and laboratorial characteristics of leprosy patients and healthy individuals.

Variables	Leprosy Cases *n* = 23 (%)	Control *n* = 20 (%)
**Gender (%)**		
Female	54	40
Male	46	60
**Age**		
20–30	55	50
31–40	28	35
41+	17	15
**Bacteriological Index**		
0	44	100
0.1–2.0	06	00
2.1–4.0	17	00
4.1–6.0	06	00
unknown	27	00
**Degree of disability**		
0	44	100
1	17	00
2	22	00
unknown	17	00
**Nerves affected**		
0	33	100
1	06	00
2	11	00
3	11	00
unknown	39	00

### Dosage of DDS

After implementation of MDT (dapsone in combination with rifampicin and clofazimine) by WHO for the treatment of leprosy in order to monitor and achieve global elimination of leprosy, some adverse reactions, mainly caused by DDS, were observed in these patients [7]. The use of dapsone may cause oxidative stress leading to an imbalance between pro-oxidant and antioxidant agents [7].

DDS mediated adverse reactions appear to be mainly due to its N-hydroxylated metabolite, DDS-NHOH and the toxicity is dose-dependent [7], [28]. Thus, the therapeutic monitoring of drugs or their metabolites is essential to promote dose adjustment in order to control the therapeutic or toxic effects [28]. In this study, data showed that MB patients after the third dose supervised (MDT3) had a plasma concentration of dapsone of 0.518±0.029 µg/mL, while PB patients had 0.662±0.123 µg/mL. There was no significant difference between the dapsone concentrations in patients with different clinical forms (p>0.05, Table 2).

**Table 2:** Dapsone plasma concentrations determined in samples of leprosy patients in multi-and paucibacillary clinical forms according to the treatment time in months.

Treatment Time (months)	DAPSONE CONCENTRATIONS (µg/mL)	
	Multibacillary	Paucibacillary
**MDT 0**	ND	ND
**MDT 3**	0,518±0.029	0,662±0.123

ND – Not detected; Samples collected before administering the first dose MDT-supervised (MDT 0) and after two months in the third dose MDT-supervised (MDT 3).

These data were similar to that reported by Vieira et al. [28], where 90% of patients presented therapeutic levels of dapsone of 0.5 to 5.0 µg/mL. Whilst some studies showed that concentrations of this drug in the plasma of leprosy patients are variable; they generally, remain within the accepted therapeutic range [7], [28]. Moreover, the values obtained in this work do not correspond to toxic concentrations. According to Carraza et al. [29], patients who had taken between 4 and 7.5 tablets of dapsone (100 mg each) had moderate to severe intoxication. In this regards, DDS levels for mild intoxication were found in patients who had average plasma concentrations up to 10 times the therapeutic level (1 µg/mL) of this drug, while 10 to 21 times (10–21 µg/mL) were moderate and over 21 times, constituted severe intoxication [30]. Although the liver is the major site of dapsone metabolism, hepatotoxicity has been observed only when the dose exceeded 300 mg/day [31].

Oral DDS is absorbed readily from the gastrointestinal tract with bioavailability of more than 86% [32]. After absorption, the drug is transported through the portal circulation to the liver, where it is metabolized via N-hydroxylation, acetylation or glucuronidation. Moreover, the peak plasma concentration after 100 mg of oral dapsone is attained between 2 to 8 hours, and 85% of it is excreted in the urine, primarily as glucuronides, and 10% in the bile [32], [33]. The long elimination half-life of dapsone averaging between 24 and 30 hours is thought to be due to several factors, such as significant enterohepatic recirculation, relatively high plasma protein binding (70–90%) of the drug and its acetylated metabolite (99%); indeed, the interconvertibility of the acetylated and parent forms also extends drug residence time [7], [34].

### Biomarkers of oxidative stress

To determine oxidative stress in leprosy patients under treatment with MDT, we evaluated the systemic levels of nitric oxide, lipid peroxidation and MetHb as indicative of damage, and also the antioxidant status through the activities of SOD and CAT, GSH levels and capacity total antioxidant by TEAC.

**Figure 2:**
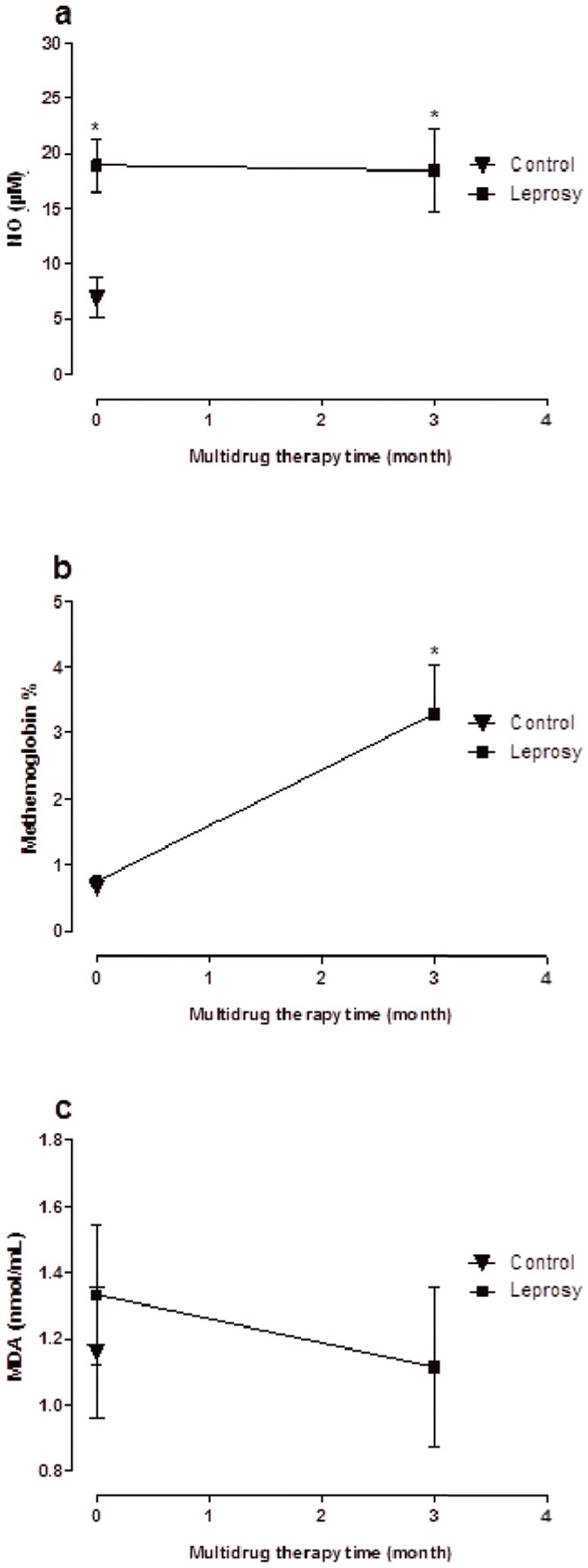
Determination of nitric oxide (NO) in serum (a), percentage of blood methemoglobin (b) levels of malondialdehyde (MDA) in plasma (c) of patients with untreated leprosy (MDT 0) and after the third dose supervised treatment (MDT 3). Figures in the chart are expressed as mean ± SD. *p<0.05 compared to MDT0 (ANOVA).

Hydroxylamines can be formed from the parent and the acetylated derivative and they are potent oxidants which cause the hematologic adverse effects associated with dapsone, including methemoglobinemia and hemolytic anemia [7], [10], [34], [35], [36]. In this regard, leprosy patients presented with significantly enhanced MetHb percentage after the third dose supervised (MDT3), with values above the normal range (<2%). Basal levels were equivalent in untreated patients (MDT0) and healthy individuals (Figure 2b). In relation to the presence of Heinz bodies, Table 3 shows that only one of the untreated leprosy patients (MDT0 group) presented with Heinz bodies, with 1 body in each 500 cells counted, while an increased detection of the bodies was seen in nine (39%) of the MDT-treated leprosy patients (MDT3 group) (two or more bodies for each 500 cells analyzed).

**Table 3:** Presence of Heinz bodies in blood smears of individuals in the study.

	HEINZ BODIES		
GROUP	Negative	Positive	Total
	*n* (%)	*n* (%)	*n* (%)
**Control**	20(100)	00(00)	20(100)
**MDT 0**	22 (96)	01(04)	23(100)
**MDT 3**	14 (61)	09(39)	23(100)

Samples collected before administering the first dose MDT-supervised (MDT 0) and after two months in the third dose MDT-supervised (MDT 3).

Methemoglobin is generated from the oxidation of the heme group of hemoglobin to the ferric state, (Fe^2+^ to Fe^3+^), and thus cannot bind oxygen, levels which exceed 70% can result in patient fatality [37]. Oxidants such as DDS metabolites as well as forming methemoglobin, also generate other reactive species, such as superoxide and hydrogen peroxide. Indeed, the intracellular oxidative stress leads to the formation of soluble and insoluble denaturation products of hemoglobin. The most apparent insoluble products include Heinz bodies [38], [39] reported that the long-term treatment only with DDS (100 mg daily) or MDT (dapsone in combination clofazimine and rifampicin) resulted in clinically significant hemolysis and MetHb, although the use of rifampicin and clofazimine, or clofazimine alone does not appear to increase the incidence of MetHb during treatment.

In agreement with our report, Dalpino et al. [40] showed that the percentage of MetHb of leprosy patients who were under treatment of dapsone (100 mg/day) was increased significantly compared to control. Regarding patient tolerance of DDS therapy, generally, patients with MetHb rates below 10% do not show any significant symptoms. However, a certain degree of hemolysis during DDS therapy appears inevitable, as alongside our present report, several previous studies have shown the presence of Heinz bodies during therapy with this drug [15], [41]. In addition, Mahmud et al. [42] reported that the dapsone (100 microM) was the most potent former of methaemoglobin in human erythrocytes, and the substitution of the sulphone group with sulphur, oxygen, nitrogen, carbon or a keto group in drug resulted in a decrease in methaemoglobin formation.

Regarding nitric oxide production, we observed that their levels were significantly higher in patients of both groups MDT0 and MDT3 when compared to healthy individuals. However, there was no difference between MDT-treated and untreated patients (Figure 2a). These data showed that the disease process appears to be responsible for the detected NO increase in the body, as shown by our previous report [43] and other studies [44], [45].

**Figure 3:**
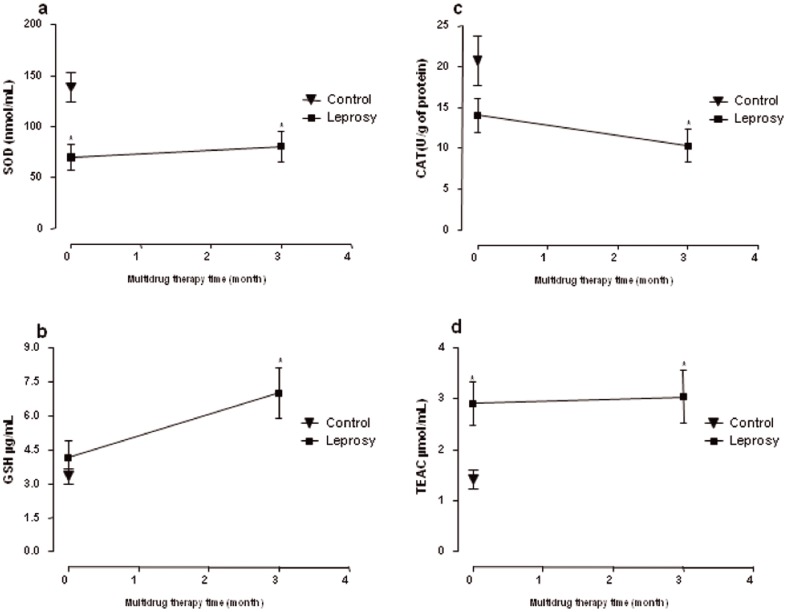
Antioxidant capacity of blood samples from untreated patients with leprosy (MDT 0) and after the third dose of supervised treatment (MDT 3). Concentration of superoxide dimutase (SOD) (**a**), reduced glutathione (GSH) (**b**), catalase (CAT) (**c**), Trolox equivalent antioxidant capacity (TEAC) (**d**). Figures in the chart are expressed as mean ± SD. *p<0.05 compared to MDT 0 (ANOVA).

Free radicals can cause cellular damage and undesired lipid peroxidation, which leads to the degradation of membrane lipid by free radicals produced by MDA. Serum/plasma levels of MDA provide some indication of the extent of f oxidative stress-related lipid peroxidation and cellular damage [46]. MDA was measured as an index of lipid peroxidation in the plasma of patients and healthy individuals. In this respect, our data showed that treatment with MDT did not alter the MDA levels in leprosy patients, as values were similar to those of healthy individuals (Figure 2c). In our report, we also observed that the MDA values in untreated or MDT-treated leprosy patients were similar to the control group (Figure 2c). These data suggest that MDT did not promote lipid peroxidation and cellular damage in the blood samples of leprosy patients. The elevated GSH contents observed in these patients may account for the normal levels of MDA in these patients after treatment with MDT (Figure 3b). In this regard, we observed similar levels between patients without treatment (MDT0) and healthy individuals. However, after treatment (MDT3 group), these patients showed significant increases in GSH values compared to the MDT0 group (Figure 3b). This protective effect of the thiol has also been demonstrated in animal models, where rats treated with 40 mg/kg of DDS yielded GSH and MetHb values significantly higher compared to control [47].

GSH is a powerful antioxidant and a cofactor in many antioxidant enzyme reactions, so an increase in GSH levels may be relevant to the regulation of the activities of antioxidant enzymes that use GSH as a cofactor and thus the maintenance of oxidative balance [48]. In addition, some studies have shown that GSH is considered a potent inhibitor of lipid peroxidation process, and thus regulates the MDA content. One of the mechanisms by which GSH performs this protective function is by regulating the activity of GSH-dependent enzymes, such as peroxidases and peroxiredoxins, which result in reduction of intracellular oxidative stress followed by inhibition of the mitochondrial pathway of apoptosis induced by ROS [49].

Concerning antioxidant enzymes, SOD activity was significantly reduced in samples from untreated leprosy patients (MDT0) and after the third dose supervised (MDT3) when compared to control group (Figure 3a). However, the basal CAT activity in untreated leprosy patients (MDT0) was similar to control group; its activity was significantly decreased by the treatment in leprosy patients (Figure 3c). The enzyme CAT is a tetrameric heme protein and mainly responsible for hydrogen peroxide degradation in aerobic and anaerobic organisms [50]. In summary, treatment with MDT led to a significant decrease in CAT activity in leprosy patients, but did not alter the SOD activity compared to untreated patients. These data were similar to other studies that reported that untreated leprosy patients have decreased levels of SOD compared to healthy individuals [51], and that even after the use of MDT, the SOD levels remained low. These findings indicate that oxidative stress related to the reduction of antioxidants and free radical increase observed in these patients may also be caused by *Mycobacterium leprae*, as reported in a previous study [43], [51]. Furthermore, studies reported that reduction of CAT activity may be associated with factors related to individuals, such as enzymatic deficiencies due to genetic mutations or a reduced synthesis of this enzyme by changes in their gene expression [52]. Many factors have been reported that can affect the gene expression of CAT, such as the presence of certain ions, cytokines and drugs [7]. In the case of infection by *M. leprae*, this agent requires ions and/or metals present in the host to regulate the expression of some of their resistance factors negatively affecting the supply of these compounds for the synthesis of metalloproteins such as CAT in the host organism [53]. On the other hand, the use of MDT has contributed to maintaining a framework of protection from oxidative stress due to host response to the infection process by *M. leprae*. In this sense, DDS metabolites possess oxidizing properties; amplifying the generation of reactive species, reducing of CAT activity and hemoglobin oxidation leading to formation of methemoglobin and Heinz bodies.

The ability of multidrug therapy to induce the production of free radicals can be compensated by antioxidant defense in leprosy patients. Antioxidants are best supplied by a balanced diet, but unfortunately leprosy patients are often of deprived socioeconomic status [54]. The analysis of the antioxidant capacity (AC) can provide some insight into the general biological antioxidant health the body, since it detects the presence of enzymatic and non-enzymatic antioxidants, instead of determining the concentrations of these antioxidants individually. In this sense, using the TEAC method for evaluation of AC has been recommended to evaluate various samples such as food, extracts and biological samples such as plasma [55]. In our study, we observed that the treatment with MDT did not alter the TEAC level in plasma of leprosy patients, which presented high levels similar to that of untreated patients (Figure 3d). Many proteins such as ceruloplasmin, transferrin, and small molecule antioxidants such as non-protein thiols, vitamins C and E, and uric acid contribute to the TEAC concentrations in plasma [56].

**Figure 4:**
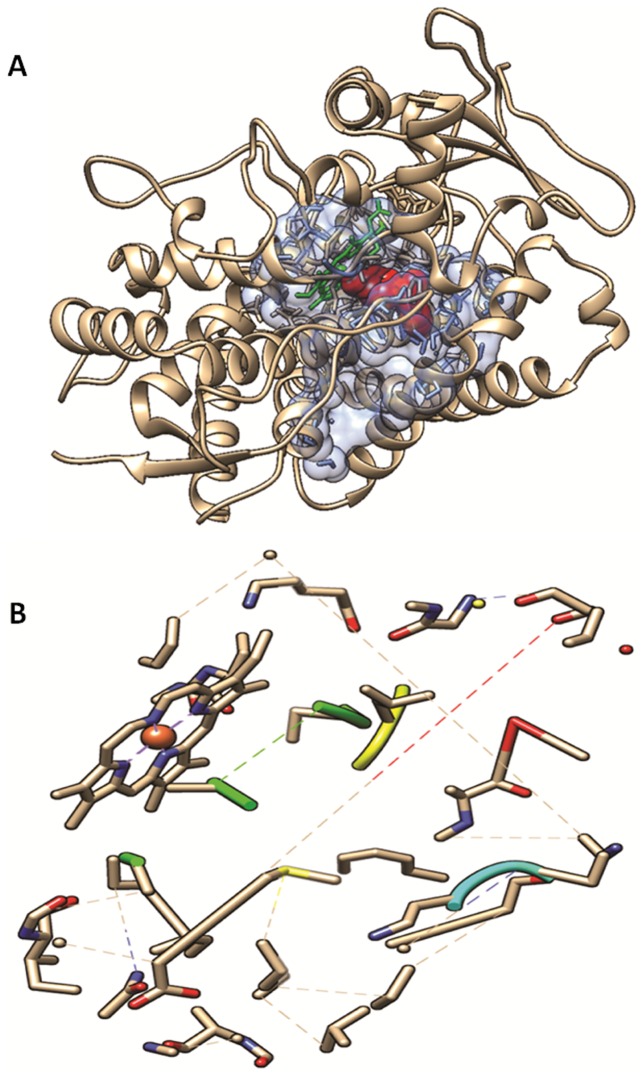
Representation of the CYP 2C19. Area covered (depicted as *light blue* transparent *solid surface*) by the two internal cavities (catalytic site cavity and adjacent cavity) of human microsomal cytochrome P450 (CYP) 2C19 (*brown*). The heme prosthetic group is represented in *green* and the molecule of dapsone in the color *red* (**a**). Delimitation of the area occupied by the two internal cavities (catalytic site cavity and adjacent cavity) of the CYP2C19 (**b**).

**Figure 5:**
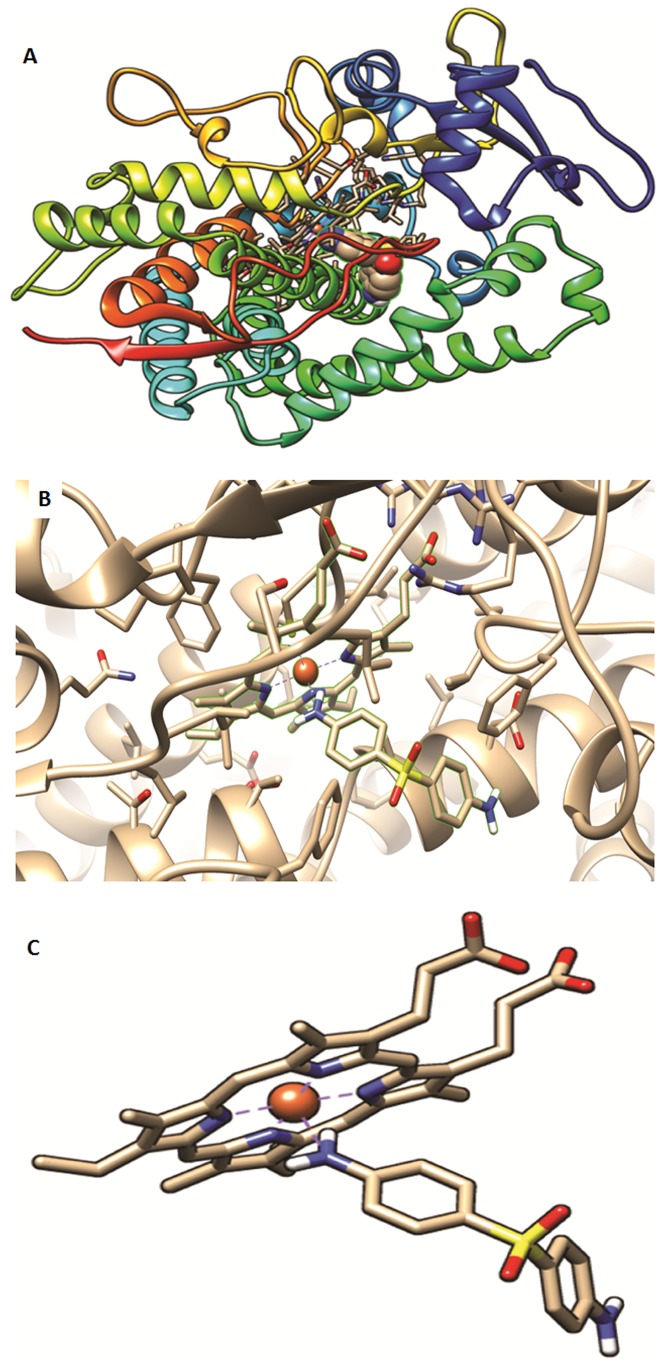
Dapsone-CYP 2C19 complex. Dapsone is shown in *spheres* and the target enzyme is colored from *blue*, starting from terminal N, through the *red*, up until terminal C (**a**). Dapsone-catalytic site CYP2C19 complex: Dapsone, the interacting amino acid residues and heme prosthetic group present in the catalytic site of CYP2C19 are displayed as *sticks*. The heme group and the dapsone are highlighted in a *green contour* (**b**). Coordination and interaction of dapsone when faced with the heme prosthetic group of the catalytic site of the CYP2C19 (**c**).

Dapsone is oxidized by a number of CYPs [57] and it is likely that several are involved in its oxidation. Although only a single report has outlined the impact of CYP2C19 on dapsone oxidation, we explored the ability of the drug to bind to this CYP isoform, which is polymorphic and has significant clinical relevance in drug clearance [58]. Analysis of the internal cavities of CYP 2C19 showed two main contiguous cavities, one acting as the catalytic site (Figure 4a), which is in agreement with those described by Reynald et al. [25]. DDS (depicted in red) binds to amino acid residues and to the heme prosthetic group in the cavity of the catalytic site, just below the heme prosthetic group, which is depicted in green (Figure 4b). These two internal cavities are associated the following characteristics: volume 1271.04 Å^3^, surface 112.44 Å^2^, lipophilic surface 801.61 Å^2^, depth 28.16 Å^2^, ellipsoid main axis c/a ratio 0.22, ellipsoid main axis b/a ratio 0.80 (Figures 4b and 5a). The characteristics of the functional groups present in the cavities in relation to their relevant forces to molecular interactions were: 19 hydrogen bond donors, 68 hydrogen bond acceptors, 1 metal, 50 hydrophobic interactions and a hydrophobicity ratio in the order of 0.36. The amino acid composition of the cavities in relation to the polarity and electrical charge was distributed as follows: nonpolar amino acids ratio 0.57, polar amino acid ratio 0.31, positive amino acids ratio 0.03 and negative amino acid ratio 0.07.

The results of the virtual molecular docking study conducted between dapsone and human microsomal CYP2C19, reveals the reactive groups present in dapsone as well as the forces and types of interactions existing between these reactive groups and the amino acid residues present in chain A of the cytochrome studied. Such data facilitates the understanding of the types of interactions and binding forces existing between them. The analysis of the interactions between dapsone and CYP 2C19 showed that DDS was located in the cavity of the reactive site, positioned near the iron atom of the heme prosthetic group (Figures 5b–c). The interactions which exist between DDS, the amino acid residues which contact it and the heme prosthetic group present in the catalytic site of CYP2C19 as well as their respective alpha-carbon positions in chain A are illustrated in Figure 5b.

**Figure 6:**
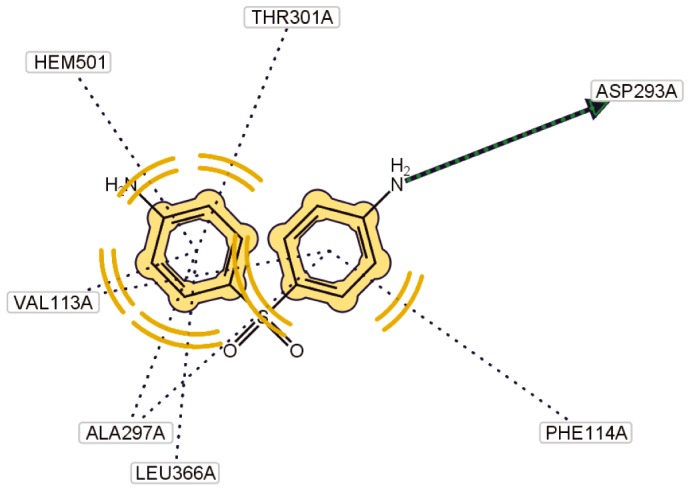
Interaction between dapsone, ligand-binding amino acid residues and the heme prosthetic group present in the catalytic site of CYP2C19 - The *arrow* represents the interaction between the hydrogen bond donating amino group (-NH2) in dapsone and the corresponding hydrogen bond acceptor (carbonyl group (CO) in residue Asp293A), dashed *orange* circular lines followed by dotted lines represent sites of hydrophobic interactions coexisting between dapsone and the respective binding residues (113A Val, Phe 114A, 297A Ala, Thr301A, Leu366A) and also with the heme prosthetic group.

The results of molecular docking analysis showed that several amino acid residues in the active site cavity interact with DDS. The main intermolecular interactions observed were the hydrophobic interactions, coexisting between dapsone and the respective amino acid residues Val113A, Phe114A, Ala297A, Thr301A and Leu366A which participate in the interactions, and also between dapsone and the heme prosthetic group, and the interaction by means of the hydrogen bond which exists between the hydrogen bond donating amino group (-NH2) in DDS and carbonyl group (CO) in amino acid residue Asp293A as the respecti8ve hydrogen bond acceptor (Figure 6). It was evident also that the amino group attached to one of the aromatic rings and positioned nearest to the heme prosthetic group was able to interact with the existing iron atom in the heme group (Figures 5b–c). Our results provide some preliminary observations on the possible DDS oxidation mechanism by CYP 2C19 for the production of DDS-NHOH.

## Conclusions

In summary, our data showed that the treatment with MDT in leprosy patients led to a decrease in enzymatic antioxidant systems as CAT, alongside an increase in GSH, as well as a rise in MetHb levels and Heinz bodies' formations. This situation may occur in consequence to an oxidant/antioxidant imbalance systemically, which is caused by a combination of the infection process and multidrug therapy in leprosy patients. The analysis of the interactions between DDS and CYP2C19 has provided some perspective for future investigations which might explore the role of this CYP in dapsone oxidation in vivo, although it is likely that the drug is cleared by multiple CYP isoforms *in vivo*.
